# Growth, ethanol production, and inulinase activity on various inulin substrates by mutant *Kluyveromyces marxianus* strains NRRL Y-50798 and NRRL Y-50799

**DOI:** 10.1007/s10295-016-1771-5

**Published:** 2016-04-29

**Authors:** Luz Ángela Galindo-Leva, Stephen R. Hughes, Juan Carlos López-Núñez, Joshua M. Jarodsky, Adam Erickson, Mitchell R. Lindquist, Elby J. Cox, Kenneth M. Bischoff, Eric C. Hoecker, Siqing Liu, Nasib Qureshi, Marjorie A. Jones

**Affiliations:** Department of Chemistry, Illinois State University, Normal, IL 61790-4160 USA; United States Department of Agriculture (USDA) Agricultural Research Service (ARS) National Center for Agricultural Utilization Research (NCAUR) Renewable Product Technology Research Unit, 1815, North University Street, Peoria, IL 61604 USA; National Coffee Research Centre (Cenicafe) National Federation of Coffee Growers of Colombia (FNC), Cenicafé Planalto Km 4 vía Antigua Chinchiná, Manizales, Caldas Colombia

**Keywords:** *Kluyveromyces marxianus*, Inulin, Inulinase, Coffee waste fermentation, Biorefinery platform

## Abstract

Economically important plants contain large amounts of inulin. Disposal of waste resulting from their processing presents environmental issues. Finding microorganisms capable of converting inulin waste to biofuel and valuable co-products at the processing site would have significant economic and environmental impact. We evaluated the ability of two mutant strains of *Kluyveromyces marxianus* (Km7 and Km8) to utilize inulin for ethanol production. In glucose medium, both strains consumed all glucose and produced 0.40 g ethanol/g glucose at 24 h. In inulin medium, Km7 exhibited maximum colony forming units (CFU)/mL and produced 0.35 g ethanol/g inulin at 24 h, while Km8 showed maximum CFU/mL and produced 0.02 g ethanol/g inulin at 96 h. At 24 h in inulin + glucose medium, Km7 produced 0.40 g ethanol/g (inulin + glucose) and Km8 produced 0.20 g ethanol/g (inulin + glucose) with maximum CFU/mL for Km8 at 72 h, 40 % of that for Km7 at 36 h. Extracellular inulinase activity at 6 h for both Km7 and Km8 was 3.7 International Units (IU)/mL.

**Electronic supplementary material:**

The online version of this article (doi:10.1007/s10295-016-1771-5) contains supplementary material, which is available to authorized users.

## Introduction

Inulin is a major storage carbohydrate present in more than 36,000 species of plants, including wheat, onion, bananas, garlic, asparagus, sunchoke, chicory, Jerusalem artichoke, dahlia, and yacón [3, 26]. In these plants, inulin is used as an energy reserve and for regulating cold resistance [36]. Inulin consists of linear chains of β-2,1-linked D-fructofuranose molecules terminating at the reducing end in a glucose residue linked by an α-1,2 bond, as in sucrose [36]. Inulinases catalyze the hydrolysis of the glycosidic linkages in inulin to produce fructose, glucose, and inulo-oligosaccharides, yielding up to 95–98 % fructose. Exo-inulinases release fructose from the fructosyl terminal, while endo-inulinases act on the internal glycosidic linkages [26]. Inulin is used as a substrate in industrial fermentation processes and in food, feed, biofuel, pharmaceutical, and chemical industries because it is a relatively cheap and abundant source for the microbiological production of ultra-high-fructose syrup, bioethanol, inulo-oligosaccharides, single-cell protein, citric acid, butanediol, and lactic acid [3, 7, 18, 27, 29, 33, 38]. The widespread occurrence of inulin in plants results in large amounts of this polysaccharide being found in fruit and vegetable waste worldwide [1]. This waste is a potential source of value-added co-products in a biorefinery platform at the fruit or vegetable processing site.

*Kluyveromyces marxianus* is a homothallic hemiascomycete yeast frequently isolated from dairy environments. It possesses phenotypic traits, such as the ability to utilize a wide range of carbohydrate substrates, secretion of lytic enzymes, notable thermotolerance and the fastest growth rate of any eukaryotic microbe, that make this yeast attractive for industrial production of ethanol from inexpensive substrates [14, 15, 17]. Because *K. marxianus* can produce both active inulinase and ethanol, inulin can be fermented directly to ethanol, and, although *K. marxianus* has poorer ethanol tolerance than *Saccharomyces cerevisiae* [3], it can tolerate ethanol concentrations of more than 100 g/L [7]. To clarify the utilization capability of sucrose, raffinose and inulin and the glucose effect on inulinase in *K. marxianus* DMKU 3-1042, Lertwattanasakul et al. [17] examined the growth and metabolite profiles of this strain on these substrates. These investigators determined that raffinose and inulin consumption was reduced by glucose at 30 °C but almost no glucose repression was observed at 45 °C. An increase in glucose concentration had no effect on sucrose utilization. These sugar-specific glucose effects were consistent with the level of inulinase activity [6, 17].investigated the effects of carbon sources, oxygenation, and ethanol on the production of inulinase by *K. marxianus* YX01. Their results showed that glucose repressed inulinase production at 30 °C, while inulin induced inulinase production. Increasing ethanol concentration repressed cell growth but did not affect activity of secreted inulinase [6]. Further studies by Gao and co-workers [7] were performed to investigate differences in gene expression between high and low inulin concentrations and from anaerobic conditions to microaeration. They determined that the differentially expressed genes were mainly associated with the pathways of central carbon metabolism and ethanol formation. Increased inulinase expression was one factor in ensuring efficient fermentation [7].

Yuan et al. [38] studied the Jerusalem artichoke (*Helianthus tuberosus*), which contains 11–20 % carbohydrates, 70–90 % of which is inulin, as an alternative feedstock for fuel ethanol production using *K. marxianus* ATCC8554. Jerusalem artichoke grows well in non-fertile land and resists many plant pests and diseases. Thus, it does not compete with grain crops for arable land and also benefits ecological environment protection. The growth and ethanol fermentation of *K. marxianus* ATCC8554 were studied using as substrate Jerusalem artichoke grown in saline soil and irrigated with a mixture of freshwater and seawater. The optimum temperatures were 38 °C for growth and inulinase production, and 35 °C for ethanol fermentation [38]. Hu and co-workers [10] investigated 87 yeast strains for inulin utilization, extracellular inulinase activity, and ethanol fermentation from both inulin and Jerusalem artichoke tuber flour at 40 °C by consolidated bioprocessing (CBP). They determined *K. marxianus* PT-1 and *S. cerevisiae* JZ1C were superior in thermotolerance and utilization of inulin-type oligosaccharides in Jerusalem artichoke tubers. It was suggested that these strains have considerable potential in ethanol production from Jerusalem artichoke tubers by high temperature CBP [10]. Kim et al. [13] showed that combining the tuber and the stalk hydrolysate is a useful strategy for whole biomass utilization in effective bioethanol fermentation from Jerusalem artichoke.

In tequila production, fermentation is an important step. Fermentation determines the ethanol productivity and organoleptic properties of the beverage. In a study by López-Alvarez et al. [19], yeast isolated from native residual agave juice from the milling process (must or wort), identified as *K. marxianus* UMPe-1, was demonstrated to be a suitable yeast for agave must fermentation, showing high ethanol productivity and increased volatile compound content compared with a *S. cerevisiae* baker’s yeast used in tequila production.

Coffee is one of the most popular beverages of the world and the second largest traded commodity after petroleum [16, 22, 24]. Large amounts of waste are generated in the coffee industry leading to serious environmental issues [2, 22, 24]. With increasing coffee production, it is imperative to apply the techniques of biotechnology in waste management to conserve both ecological and economical resources [22, 25]. Advances in industrial biotechnology offer potential opportunities for economic utilization of coffee industry waste [28]. The main residues of coffee processing are the husks, pulp, mucilage, parchment, and silverskin, comprising 45 % of the coffee fruit [5]. Coffee pulp contains about 13 mg fructans/g and coffee mucilage contains about 35 mg fructans/g [12]. These materials are ideal substrates for microbial processes for the production of value-added products [23, 26].

We were interested in evaluating *K. marxianus* mutant strains NRRL Y-50798 (Km7) and NRRL Y-50799 (Km8) [11] for potential use in fermentation of inulin-containing food and plant waste as part of an integrated biorefinery platform. Both of these mutant strains were studied for growth and utilization of chicory inulin (I) in four media, YPD, YPI, YPDI, and 1 % Inulin (see Table 1 for composition). Growth was measured by determining colony forming units (CFU)/mL. Concentrations of glucose and fructose were measured to determine consumption of glucose or degradation of inulin to fructose. Ethanol production by the mutant strains was also determined in these four media. In addition, inulin extracted from crude coffee processing waste (skin, pulp, and mucilage) was studied as a substrate for ethanol production. Finally, the level of inulinase activity in these mutant strains was determined.

**Table 1:** Composition of media used in fermentation experiments

Component (g/L)	YPD	YPI	YPDI	1 % Inulin
Yeast	10	10	10	–
Peptone	20	20	20	–
Glucose	20	–	10	–
Inulin	–	20	10	10

## Materials and methods

### Strains

*K. marxianus* mutant strains NRRL Y-50798 (Km7) and NRRL Y-50799 (Km8) were derived from *K. marxianus* NRRL Y-1109 cultures (USDA, ARS Culture Collection) by UV-C irradiation followed by 5 month growth on glucose at 46 °C under anaerobic conditions [11]. The strains are optimized for growth at elevated temperature under microaerophilic conditions.

### Inulin media composition and preparation

Four media, YPD, YPI, YPDI, and Inulin, containing different proportions of inulin from chicory (Sigma-Aldrich, St. Louis, MO), sugar (dextrose; Fisher Scientific, Fair Lawn, NJ), yeast (Bacto™ Yeast Extract; Becton, Dickinson and Company, Sparks, MD), and protein (peptone; Bacto™ Peptone, Becton, Dickinson and Company, Sparks, MD) were prepared using nanopure water. Media were sterilized by autoclaving for 15 min at 250 °F and 20 psi (Amsco Renaissance Series 3021 Gravity). The composition of each medium used is shown in Table 1.

### Inoculum preparation

Three colonies of *K. marxianus* mutant strain, either Km7 or Km8, used within 15 days of plating on YPD 2 % w/v agar (Bacto™ Agar, Becton, Dickinson and Company, Sparks, MD), were inoculated into a 250 mL flask containing 60 mL of YPD medium and incubated at 30 °C with constant shaking at 100 rpm for 48 h. After 48 h of incubation, the absorbance values at 600 nm for Km7 and Km8 cultures were 1.43 and 1.38, respectively (Hewlett Packard UV/Visible spectrophotometer with ChemStation Software). Therefore, the amounts of inoculum taken to initiate the fermentations were 350 µL of the Km7 liquid culture and 363 µL of the Km8 culture so that the amount of cells initially used was the same for both strains.

### Fermentation of inulin mixtures

Sterile 250 mL shake flasks containing 60 mL of each medium were inoculated with a culture of either strain Km7 or Km8 grown for 48 h, with the inoculum volume adjusted to contain the same number of cells for each strain. A negative control with no cell culture added was used for each medium to detect changes in concentration of any media components whose consumption or production was being investigated. All cultures were incubated at 30 °C for 96 h at 100 rpm. At 0, 1, 2, 4, 8, 24, 48, 72 and 96 h, a 2 mL sample was taken from each flask, 1 mL for growth determination and 1 mL for high performance liquid chromatography (HPLC) analysis.

### Growth determination

To determine the growth of the *K. marxianus* strains tested, 1 mL samples from each time point were diluted 10^2^-fold and 10^4^-fold, and spread onto petri dishes containing YPD agar. The dishes were incubated at 30 °C for 24–48 h, and the CFUs were counted after at least 24 h of growth.

### Determination of glucose, fructose and ethanol concentrations

At each time point, 1 mL samples obtained from each fermentation flask were cleared by centrifugation at 14,000 ×*g* for 15 min, and 100 µL of supernatant were taken, added to 900 µL deionized water, and stored at −80^o^ C until analyzed. Glucose, fructose, and ethanol concentrations were determined at each time point using an HPLC separation system consisting of a solvent delivery system (P2000 pump, Spectra-Physics, San Jose, CA) equipped with an autosampler (717, Waters Chromatography Division, Millipore Corp., Milford, MA) and a computer software based integration system (Chromquest 4.0, Spectra-Physics). An ion moderated partition chromatography column (Aminex HPX 87H with Cation H micro-guard cartridge; Bio-Rad Laboratories, Inc., Hercules, CA) was used. Samples (10 µL) were injected onto a heated column (65 °C), and eluted at a flow rate of 0.6 mL/min with 5 mM H_2_SO_4_. Peaks were detected with a refractive index detector (410 differential refractometer, Waters Chromatography Division, Millipore Corp., Milford, MA) and were identified and quantified by comparison to retention times of authentic standards [4, 32].

### Thin layer chromatography (TLC) determination of glucose, fructose, and inulin

TLC analyses to determine the presence of fructose, glucose, and inulin in cell-free culture supernatants from cultures of strains Km7 and Km8 in 1 % inulin were performed at 2, 24, 48, 72 and 96 h using 20 × 20 cm silica gel 60F254 thin layer chromatography plates (EMD Chemicals). Samples were cleared by centrifugation at 10,000 ×*g* for 5 min. A total of 6 µL of supernatant was loaded (2 µL aliquots at a time) into separate lanes on the plates. A control of 1 % inulin incubated in the absence of either yeast strain, to evaluate stability of inulin during incubation, and standard solutions of fructose, glucose, and inulin were also applied. A solvent mixture of 1-butanol, 2-propanol, water, and acetic acid (7:5:4:2 v/v/v/v) was employed following the method of Tomita [34]. Spots were visualized by spraying the plate with 4 M H_2_SO_4_ in methanol and heating it at low temperature on a hot plate.

### Crude inulin extraction from coffee waste (CW-I)

Crude inulin (CW-I) was extracted from milled lyophilized coffee processing waste, consisting mainly of skin, pulp, and mucilage from the coffee berry (Cenicafe, Colombia, South America), with hot water (80 °C) for 1 h using the general method of Mavumengwana [20]. The inulin extract from chicory root obtained using this method contained 80 % inulin and 4 % free fructose [20]. Coffee waste (pulp plus mucilage) has been shown to contain 4.8 % fructans in addition to other sugars and protein that would likely be extracted using this method [12]. 75 mL of CW-I extract were mixed with 75 mL of nanopure water and sterilized by autoclave as described above for medium preparation. 20 mL of the sterile CW-I mixture were transferred into 50 mL sterile polypropylene blue screw-top test tubes (Fisher Scientific, Fair Lawn, NJ). The CW-I fermentation experiments using mutant strains Km7 and Km8 were carried out in the 50 mL tubes.

### Inoculum preparation and fermentation of coffee-waste inulin

A starter culture was created for both yeast strains (Km7 and Km8) for the CW-I fermentation. The starter cultures were grown aerobically for 48 h at room temperature. For fermentation, 500 µL of the Km7 and Km8 starter cultures were used to inoculate 20 mL of CW-I in each of two 50 mL fermentation tubes. These were then grown aerobically at 30 °C with shaking at 150 rpm in a New Brunswick Scientific Co Inc. Series 25 Incubator Shaker. During the experiment, a 2 mL sample of each of the CW-I fermentations was taken at 0, 6, 12, 24, 30, 48, 72 h. One mL was frozen at −80 °C to use for GC determination of ethanol production, while the other 1 mL was analyzed for cell growth. To determine growth, 10 µL of the fermentation broths were diluted 10^2^-fold and 10^4^-fold, spread with a sterile cell spreader onto petri dishes containing YPD agar, and incubated for 24–48 h at 30 °C before CFUs were determined.

### Detection of ethanol by gas chromatography

A Thermo Scientific Focus GC-FID with an auto-sampler (AS3000) was used for detection of ethanol production. A set of ethanol standards were prepared to determine the concentration of ethanol produced during the fermentation. A 5 % (v/v) *n*-propanol internal standard was added to each of the standards and samples to determine reproducibility of instrument response. Stored frozen samples were thawed and the cells pelleted by centrifugation. Then 500 µL of the supernatant were transferred to a GC vial and 450 µL of nanopure water and the internal standard (50 µL) were added for a final volume of 1 mL. Duplicate injection volumes of 0.7 µL were used; the inlet and detector were set to 250 °C. The carrier gases, helium (175 kPa), compressed air (220 kPa), and nitrogen (500 kPa), were at a constant flow of 1.2 mL/min. Each GC separation was run for 2.5 min at 75 °C.

### Zymogram assay

The activity of secreted inulinase from mutant strains Km7 and Km8 was evaluated using a modified zymogram procedure [8]. An agarose (1 % w/v) suspension with chicory inulin (0.1 % w/v; Sigma-Aldrich, St. Louis, MO) in nanopure water was heated in a microwave and poured into a Bio-Rad Sub-Cell horizontal tray (10 × 15 cm). After cooling to room temperature, the gel was cut into pieces 50–55 mm on each side, and each piece was placed into a 100 mm petri dish. A cell suspension of each of the strains was prepared by placing several colonies obtained from YPD agar plates into 1 mL of sterile nanopure water and mixing gently. The suspension was applied to the inulin-containing gels using a 10 µL inoculation loop. Negative controls were inulin gel pieces with no suspension added. The gels were incubated at 30 °C for 5 days and observed for growth. A Sigma-Aldrich Periodic Acid-Schiff (PAS) staining kit (Sigma-Aldrich, St. Louis, MO) was used according to instructions to stain the gels. The periodic acid solution was added to the petri dish and swirled for 30–60 s to allow complete coverage of the gel. Excess solution was decanted and the Schiff reagent was added and swirled in the same manner. When treated with periodic acid, glycols are oxidized to aldehydes. After reaction with Schiff’s reagent (a mixture of pararosaniline and sodium metabisulfite), a pararosaniline adduct is released that stains the glycol-containing cellular elements pink to red or violet. The PAS system produces a pink color with inulin (Sigma-Aldrich). Absence of pink color in the inulin-containing gel indicates degradation of the inulin. The gel was observed for presence or absence of a pink color.

### Quantitation of inulinase activity

Quantitative data for inulinase activity of mutant strains Km7 and Km8 were obtained by HPLC analysis of fructose formed using cultures of the mutant strains compared to standard solutions of inulinase (endohydrolase from *Aspergillis niger*; EC Number 3.2.1.7; 240 IU/mL; Sigma-Aldrich, St. Louis, MO) with 0.2 % chicory inulin as the substrate. The activity of inulinase from *A. niger* is similar to that from *K. marxianus*, for example, using inulin from dahlia tuber extract as substrate, inulinase activity for *A. niger* was 2–4 U/mL and for *K. marxianus* was 1.5 U/mL [31]. Four standard inulinase solutions were each prepared in triplicate by adding 10 µL of undiluted, 10-, 100-, and 1000-fold dilutions of the standard inulinase solution to 40 µL of 50 mM sodium phosphate buffer (pH 7) and adding the solution to 850 µL of sterile water in a microfuge tube, followed by addition of 100 µL of inulin (20 mg/mL) to the tube [21 modified]. Suspensions (no dilution and 10-fold dilution) of 2 day 30 °C cultures of strains Km7 and Km8 adjusted to OD = 2 at 600 nm were prepared similarly. The tubes were capped and incubated at 30 °C for 2, 4, and 6 h. At each time point, the tubes were removed from the incubator and placed in a water bath at 90 °C for 10 min to inactivate the enzyme (inulinase denatures at 77 °C [30]). The amount of fructose formed in the solutions was measured using HPLC. The inulinase activity versus the amount of fructose released (area under fructose HPLC peak) was plotted for the standards (data not shown). The inulinase activities in Km7 and Km8 were determined from the standard plots using the area under the fructose peaks in the Km7 and Km8 reactions.

## Results

### Growth levels

**Fig. 1:**
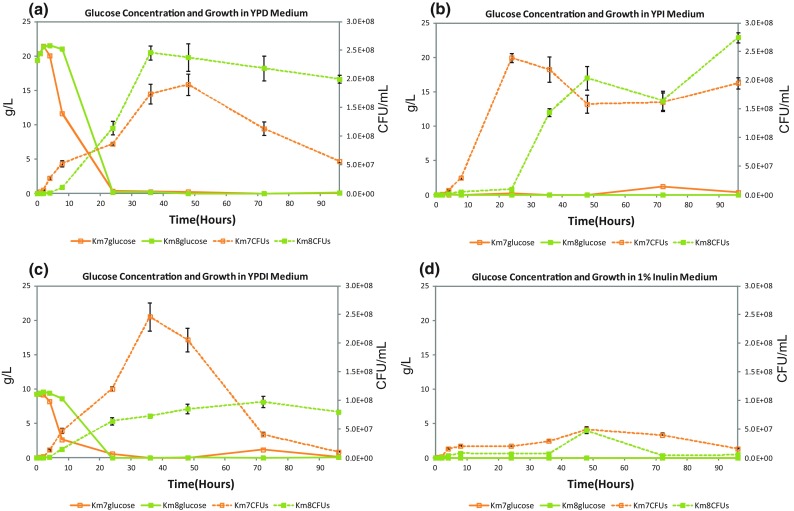
Glucose concentrations and cell growth as a function of incubation time with mutant strains Km7 and Km8 using 4 different media: **a** YPD, **b** YPI, **c** YPDI, and **d** 1 % inulin

**Fig. 2:**
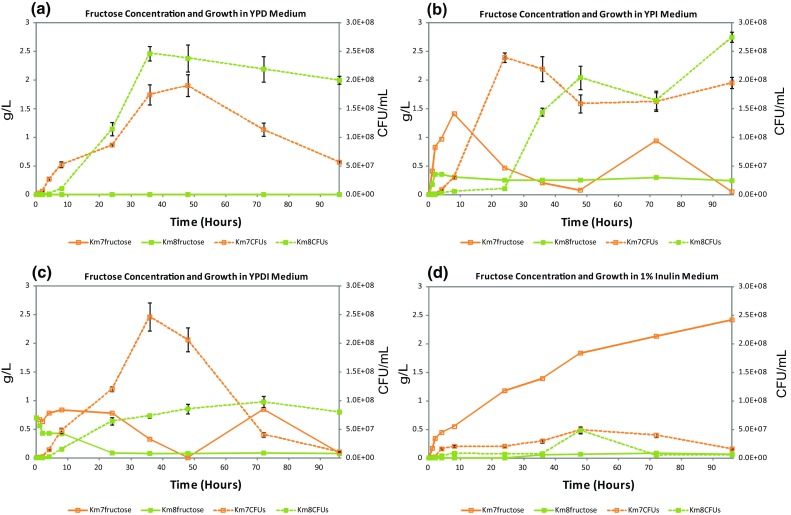
Fructose concentrations and cell growth as a function of incubation time with mutant strains Km7 and Km8 using 4 different media: **a** YPD, **b** YPI, **c** YPDI, and **d** 1 % inulin

**Fig. 3:**
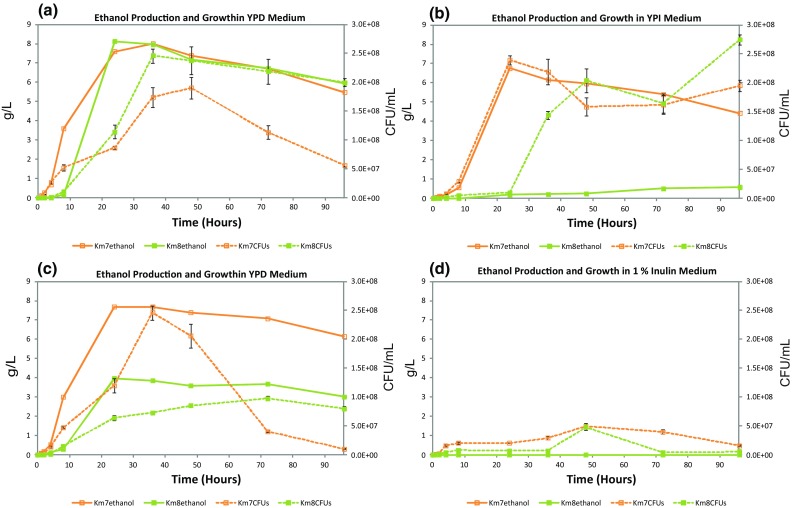
Ethanol production and cell growth as a function of incubation time with mutant strains Km7 and Km8 using 4 different media: **a** YPD, **b** YPI, **c** YPDI, and **d** 1 % inulin

Growth levels (CFU/mL) as a function of incubation time for mutant strains Km7 and Km8 with 4 different media, YPD, YPI, YPDI, and 1 % chicory inulin, are shown in Figs. 1–3a–d, respectively. For mutant strain Km7, the most favorable media for growth were YPI and YPDI, in both of which Km7 showed maximum growth of about 2.5 × 10^8^ CFU/mL at 24 and 36 h, respectively (Fig. 1b, c). YPI medium (Fig. 1b) appeared to sustain growth over a longer time relative to YPD and YPDI media. The maximum growth of Km7 in YPD medium was 1.9 × 10^8^ CFU/mL at 48 h (Fig. 1a). Growth of Km7 in 1 % (w/v) chicory inulin medium reached a maximum of 5.0 × 10^7^ CFU/mL at 48 h, then decreased to a sustained level of about 2.0 × 10^7^ CFU/mL up to 96 h (Fig. 1d).

Mutant strain Km8 demonstrated greatest growth in YPD and YPI media, with the maximum of 2.5 × 10^8^ CFU/mL occurring at 36 h in YPD medium (Fig. 1a) and a maximum of 2.7 × 10^8^ CFU/mL occurring at 96 h in YPI medium (Fig. 1b). In YPI medium, in contrast to Km7 where the growth maximum was at 24 h with a slow decrease to about 2.0 × 10^8^ CFU/mL at 96 h, Km8 showed a lag time of 24 h before growth started, with an initial growth peak of 2.0 × 10^8^ CFU/mL observed at 48 h, a decrease to 1.6 × 10^8^ CFU/mL at 72 h, and an increase to 2.7 × 10^8^ CFU/mL (and still rising) at 96 h (Fig. 1b). In YPDI medium, maximum growth of Km8 was 1.0 × 10^8^ CFU/mL at 72 h (Fig. 1c) about 40 % that of Km7 in YPDI at 36 h (or of Km8 in YPD at 36 h). In 1 % inulin medium, growth of Km8 reached a maximum of 5.0 × 10^7^ CFU/mL at 48 h dropping to an undetectable level at the remaining time points (Fig. 1d). The growth results are summarized in Table 2.

**Table 2:** Comparison of growth, substrate utilization, and ethanol production with *K. marxianus* mutant strains Km7 and Km8 in YPD, YPI, YPDI, and 1 % inulin media to *K. marxianus* strains in the literature

Strain	Medium/temp.	Maximum growth	Glucose (g/L)	Fructose (g/L)	Inulin (g/L)	Maximum ethanol produced	Grams ethanol/grams substrate	References
Km7	YPD [2 % (w/v) glucose]/30 °C	1.9 × 10^8^ CFU/mL at 48 h	20 g/L to 0 g/L by 24 h (20 g/L used)	None detected	None added	8.0 g/L at 36 h	0.40 g EtOH/g glucose	This study
Km8		2.5 × 10^8^ CFU/mL at 36 h	20 g/L to 0 g/L by 24 h (20 g/L used)	None detected	None added	8.0 g/L at 24 h	0.40 g EtOH/g glucose	
Km7		OD_600_ = 0.067	44 g/L used	None	None added	20.0 g/L at 72 h	0.45 g EtOH/g glucose	[11]
Km8		OD_600_ = 0.190	44 g/L used	None	None added	22.5 g/L at 72 h	0.51 g EtOH/g glucose	
Km7	YPI [2 % (w/v) inulin]/30 °C	2.4 × 10^8^ CFU/mL at 24 h	Negligible except for small peak of 1.2 g/L at 72 h	Peak 1.4 g/L at 8 h; 0 at 48 h; 0.9 g/L at 72 h	20 g/L added^a^	7 g/L at 24 h	0.35 g EtOH/g inulin	This study
Km8		2.7 × 10^8^ CFU/mL at 96 h	None detected	0.4 g/L at 4 h levels off at 0.3 g/L 8-96 h	20 g/L added^a^	0.6 g/L at 96 h	0.03 g EtOH/g inulin	
DMKU 3-1042 (static)		OD_660_ = 10.5 at 36 h (10.0 at 24 h)	None	0 to 3 g/L at 6 h to 0 at 24 h	17 g/L to 0 g/L by 36 h (17 g/L used)	9 g/L at 48 h	0.53 g EtOH/g inulin	[17]
Km Y179	YPI (120 g inulin in YP)/30 °C	OD_620_ = 5.5 at 16 h	114 g/L total sugars used at 24 h		(peak of 50 g/L at 4 h)	51 g/L at 24 h	0.45 g EtOH/g total sugars	[7]
Km Y179	Dry tuber meal added to reactor	High gravity	106 g/L total sugars used at 48 h		(50 g added at 24 h)	71 g/L	0.67 g EtOH/g total sugars	[37]
Sc with Km inulinase gene	Inulin added to reactor				188.2 g/L in bioreactor; converted at 48 h	80.2 g/L	0.43 g EtOH/g inulin	[9]
Km7	YPDI [1 % (w/v) glucose; 1 % (w/v) inulin]/30 °C	2.5 × 10^8^ CFU/mL at 36 h	10 g/L to 0 g/L by 36 h (10 g/L used)	0.7 to 0.8 g/L at 8-24 h to 0 at 48 h; up to 0.8 at 72 h	10 g/L added^a^	7.7 g/L at 24 h	0.39 g EtOH/g (glu + in)	This study
Km8		1.0 × 10^8^ CFU/mL at 72 h	10 g/L to 0 g/L by 24 h (10 g/L used)	0.7 to 0.4 g/L at 0-8 h to negligible from 24-96 h	10 g/L added^a^	4.0 g/L at 24 h	0.20 g EtOH/g (glu + in)	This study
DMKU 3-1042(static)	YPDI [2 % (w/v) glu; 2 % (w/v) inulin]/30 °C	OD_660_ = 14.5 at 48 h(14.0 at 36 h)	17 g/L to 0 g/L by 18 h (17 g/L used)	2 to 3 g/L at 6-24 h back to 0 at 48 h	19 g/L to 0 g/L by 48 h (19 g/L used)	17 g/L at 48 h (still rising)	0.47 g EtOH/g (glu + in)	[17]
Km7	1 % inulin/30 °C	5.0 × 10^7^ CFU/mL at 48 h (rest 2.0 × 10^7^)	None detected	Steady rise 0 to 2.4 g/L at 2-96 h (still rising at end)	10 g/L added^a^	None detected	–	This study
Km8		5.0 × 10^7^ CFU/mL at 48 h (rest 0)	None detected	None detected 0-24 h; < 0.1 g/L 24-96 h	10 g/L added^a^	None detected	–	This study

^a^Concentration not measured by HPLC during experiment

### Glucose, fructose, and ethanol concentrations determined by HPLC

Glucose concentrations (g/L) as a function of incubation time using mutant strains Km7 and Km8 with 4 different media, YPD, YPI, YPDI, and 1 % chicory inulin, are shown in Fig. 1a–d, respectively. Growth levels (CFU/mL) as a function of time are also presented. In YPD and YPDI media for both the Km7 and Km8 mutant strains, glucose is essentially completely utilized by 24 h, with maximum growth occurring at approximately 36 h for Km7 in both media and for Km8 in YPD (Fig. 1a, c). However, Km8 in YPDI, although nearly all glucose was consumed at 24 h (concentration was not completely to baseline until 36 h; Fig. 1c), exhibited only a gradual increase to a relatively low maximum growth at 72 h (40 % of that in YPD). In YPI and 1 % inulin media (Fig. 1b, d), glucose is essentially not detected for either strain except for a small peak (0.5 g/L) at 72 h for Km7 in YPI. Both Km7 and Km8 strains grow well in YPI medium, but, although maximum growth levels are similar for both strains, for Km7 the maximum occurs at 24 h, while for Km8 it occurs at 48 h (Fig. 1b). In 1 % inulin medium (Fig. 1d), no glucose is detected (only one line is shown), and the maximum growth at 48 h of Km7 was 20 % and of Km8 was 12 % of that for these strains in YPI.

Fructose concentrations (g/L) and cell growth (CFU/mL) as a function of incubation time using mutant strains Km7 and Km8 with the 4 different media, YPD, YPI, YPDI, and 1 % chicory inulin, are presented in Fig. 2a–d, respectively. Note that the scale on the *left y-axis* denoting sugar concentration is different on Figs. 1 and 2, while the scale on the right y-axis representing cell growth is the same on all figures. No fructose is detected in YPD medium at any time point (only one line is shown) with either Km7 or Km8 (Fig. 2a). In YPDI medium, fructose is present for both strains, with Km7 reaching a maximum of about 0.8 g/L at 8–24 h, dropping to an undetectable level at 48 h, then returning to about 0.8 g/L at 72 h, and with Km8 starting at 0.7 g/L at 2 h and decreasing to a negligible level (< 0.1 g/L) by 24–96 h (Fig. 2c). In YPI medium both strains show somewhat similar fructose patterns to those they displayed in YPDI, with fructose concentration for Km7 reaching a maximum of about 1.4 g/L at 8 h, dropping to a negligible level at 48 h, returning to about 0.9 g/L at 72 h, then dropping to negligible at 96 h, and for Km8 reaching a maximum of 0.4 g/L at 4 h and leveling off to about 0.3 g/L by 24 h and continuing at that level to 96 h (Fig. 2b). In the 1 % inulin medium, the fructose concentration in the Km7 culture increases steadily from < 0.1 g/L initially to 2.4 g/L at 96 h, while the fructose concentration in the Km8 culture is negligible (< 0.1 g/L) from 0 to 96 h (Fig. 2d).

Ethanol production (g/L) and cell growth (CFU/mL) as a function of incubation time using mutant strains Km7 and Km8 with four different media, YPD, YPI, YPDI, and 1 % inulin, are shown in Fig. 3a–d, respectively. In YPD medium (Fig. 3a), ethanol production was similar for the two strains (maximum of about 8.0 g/L at approximately 24 h), although Km8 achieved slightly higher maximum growth (2.5 × 10^8^ CFU/mL at 36 h) than Km7 (1.9 × 10^8^ CFU/mL at 48 h). In YPDI medium (Fig. 3c), ethanol production for Km7 followed the same pattern as in YPD medium; however, ethanol production for Km8 in YPDI medium was much lower, with a maximum of about 4 g/L at 24 h, half that of Km8 in YPD medium, correlating with the reduced growth of Km8 in YPDI medium (maximum 1.0 × 10^8^ CFU/mL at 72 h), about 40 % that of Km8 in YPD medium. In YPI medium (Fig. 3b), maximum growth with Km8 was similar to that with Km7, although the maximum for Km8 was at 96 h while that for Km7 was at 24 h, earlier than in YPD and YPDI media. However, Km8 produced a negligible amount of ethanol, while Km7 produced the same maximum amount of ethanol as in YPD and YPDI media (about 8 g/L) but, notably, the maximum appeared about 12 h earlier in YPI medium coinciding with maximum growth. When grown in 1 % inulin medium, neither strain produced ethanol (only one line is shown); however, Km7 exhibited sustained low level growth (2 to 5 × 10^7^ CFU/mL, maximum at 48 h) while Km8 produced a small growth peak reaching 5.0 × 10^7^ CFU/mL above baseline at 48 h (Fig. 3d). The glucose, fructose, and ethanol results are summarized in Table 2.

### Thin layer chromatography results

**Fig. 4:**
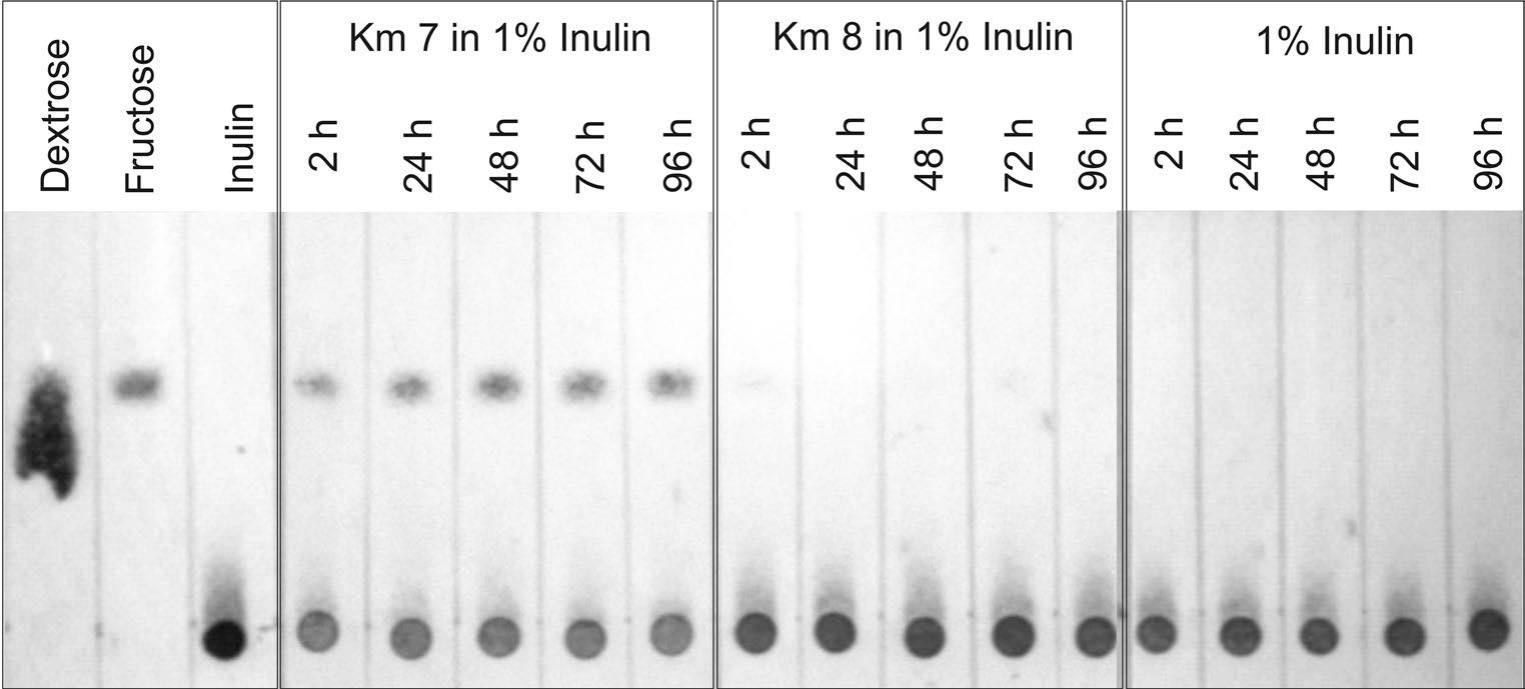
Thin layer chromatography determination of glucose (dextrose), fructose, and inulin; *panel 1*: standard glucose, fructose, and inulin solutions; *panels 2* and *3*: mutant strains Km7 and Km8 incubated in 1 % inulin; panel 4: 1 % inulin incubated without either strain added

Thin layer chromatography results of samples taken at 2, 24, 48, 72, and 96 h during the incubation of mutant strains Km7 and Km8 in 1 % inulin medium are presented in Fig. 4. The chromatogram for Km7 in 1 % inulin indicated production of fructose with a decrease in the amount of inulin. The chromatogram for Km8 showed the presence of inulin with no significant degradation to fructose. The chromatogram for a control sample of 1 % inulin medium incubated for 96 h without added yeast is also provided in Fig. 4, indicating no degradation of inulin occurred in the absence of the yeast strains.

### Fermentation of crude inulin extract from coffee waste (CW-I)

**Fig. 5:**
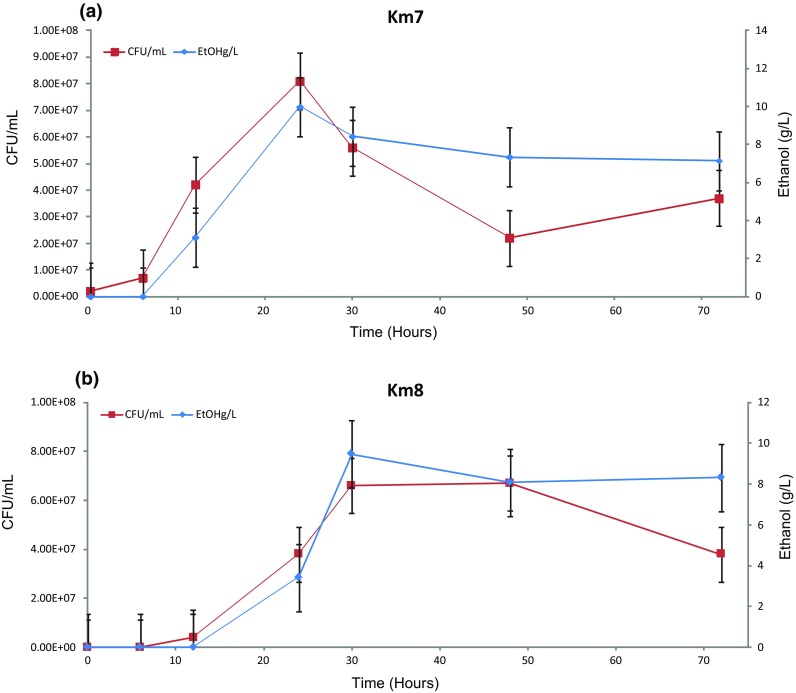
Ethanol production (g/L) and cell growth (CFU/mL) for mutant strain Km7 **a** or Km8 **b** in medium with crude inulin extracted from coffee processing waste

Fermentation of crude inulin from coffee processing waste by mutant strains Km7 or Km8 is shown in Fig. 5a–b, respectively. The growth (CFU/mL) and the amount of ethanol (g/L) detected are shown as a function of time. Mutant strain Km7 reached a maximum growth of 8.0 × 10^7^ CFU/mL at 24 h, while mutant strain Km8 reached a maximum growth of 6.0 × 10^7^ CFU/mL at 30 h. For both strains, maximum growth was correlated with maximum production of ethanol. For Km7 maximum ethanol production was 10 g/L and for Km8 it was 9 g/L.

### Demonstration of inulinase activity

Zymogram analysis was used to demonstrate inulinase activity by the mutant yeast strains. An aqueous suspension of Km7 or Km8 colonies grown on YPD agar was applied to an agarose gel containing 0.1 % (w/v) inulin and incubated for 5 days at 30 °C. When periodic acid solution and Schiff’s reagent were added to the negative control inulin gel and the color allowed to develop, the entire surface of the gel was an even pink color. On the gel containing mutant strain Km7, which occupied a circular region of growth about 2.5 cm in diameter, a colorless ring (halo) about 5 mm wide evenly surrounding the entire circumference of the region of cell growth was observed, while the gel beyond the colorless ring was pink. The area of cell growth was stained purple. For mutant strain Km8 the colorless ring was about one-third the width of that for mutant strain Km7 (data not shown). The results for Km7 are depicted in Figure A1 in the Supplementary Electronic Information.

### Quantitation of inulinase activity

**Fig. 6:**
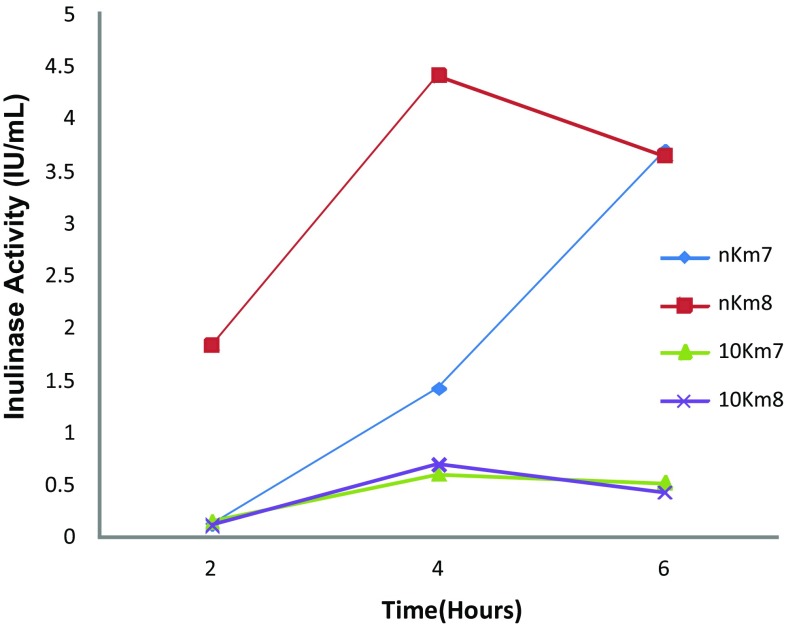
Inulinase activity (International Units (IU)/mL) of mutant strains Km7 and Km8, using HPLC to measure the amount of fructose (area under peak) produced from incubation of cell cultures at 30 °C with 0.2 % inulin solution at 2, 4, and 6 h by these strains compared to a standard inulinase solution. Key: n Km7 = undiluted Km7 culture; n Km8 = undiluted Km8 culture; 10 Km7 = 10-fold dilution of Km7 culture; 10 Km8 is10-fold dilution of Km8 culture

Results quantitating inulinase activity of mutant strains Km7 and Km8, using HPLC to measure the amount of fructose (area under peak) produced from 0.2 % inulin at 2, 4, and 6 h by these strains compared to a standard inulinase solution, are provided in Fig. 6. Extracellular inulinase activity for undiluted Km7 strain was 0.13 IU/mL at 2 h, rose to 1.4 IU/mL at 4 h and continued to rise to 3.7 IU/mL at 6 h. Extracellular inulinase activity at 2 h for undiluted Km8, 1.83 IU/mL, was higher than that for Km7. The level for Km8 increased to a maximum of 4.4 IU/mL at 4 h and then decreased to 3.7 IU/mL at 6 h. Km7 at its maximum at 6 h was at the same level as Km8 at 6 h, but its activity appeared to be continuing to increase unlike that of Km8, which was decreasing at that point. Extracellular inulinase activity of a 10-fold dilution of Km7 followed a similar pattern to that of Km8, reaching a similar maximum at 4 h, 0.60 IU/mL for Km7 and 0.69 IU/mL for Km8. Both mutant strains gave secreted inulinase activity levels comparable to those for *K. marxianus* reported in the literature on various inulin substrates. Rawat et al. [31] obtained inulinase activity values at 72 h for *K. marxianus* MTCC 3995 of 0.41 U/mL on asparagus root extract and of 1.49 U/mL on dahlia tuber extract. Yuan and co-workers [37] determined values of 3–4 U/mL for inulinase activity of *K. marxianus* Y179 between 12 and 48 h in fermentation experiments using Jerusalem artichoke tuber meal.

## Discussion

Both *K. marxianus* mutant strains used in this study grew well in YPD medium utilizing all available glucose by 24 h and producing ethanol in amounts comparable to the amounts previously obtained with these strains [11]. Maximum growth in YPD was slightly higher and earlier for Km8 than Km7. Maximum ethanol production correlated with the depletion of glucose at 24 h for Km7 and Km8. Ethanol yield was the same for both strains (0.40 g ethanol/g glucose). In YPI medium, both strains grew well, but the Km7 cells reached maximum growth sooner than Km8, possibly the result of more rapid production of fructose, with the maximum growth peak for Km7 at 24 h compared to 96 h for Km8 suggesting Km7 was able to hydrolyze inulin or induce inulinase more readily than Km8. The maximum concentration of fructose in YPI occurred at 8 h for Km7, but the level of fructose for Km8 never rose as high and remained much lower throughout the 96 h. For Km7 in YPI, the rise in CFU/mL at 72 h following the drop in growth after maximum growth corresponds to the second fructose peak at 72 h, which is possibly related to inulinase induction. The release of fructose may explain the increase in CFU/mL at that time. Most reports in the literature show complex behavior of inulinases on inulin substrates for a variety of reasons [20], but a similar drop after maximum growth followed by a rise in cell growth has been reported [7, 20]. However, the same explanation does not apply to the similar rise in cell growth for Km8. Although Km7 and Km8 showed the same inulinase activity level at 6 h, Km8 in YPI at 72 h did not release fructose at the same time or in the same amount as Km7, so the reason for the rise in growth for Km8 is not clear. It may possibly be linked to a difference between the two strains in the induction of inulinases, but further assessment is needed. It was of interest to note that with the Km7 cells, glucose was detectable at 72 h (although glucose was not added to the medium). We speculate that enzymes in Km7 release the small amount of glucose present at the inulin polymer termini linked through a sucrose bond. In YPDI medium with Km8, the amount of glucose was reduced to undetectable levels by 24 h of incubation similar to Km8 in YPD medium, while with Km7 in YPDI medium, glucose was not completely depleted until 36 h (although most was consumed by 24 h) in contrast to Km7 in YPD medium. For both strains the fructose concentration trends were similar in YPI and YPDI media. The maximum growth of Km7 in YPDI medium was slightly higher than that of Km7 in YPI medium; however, although Km8 in YPDI consumed nearly all available glucose by 24 h, this strain exhibited only a gradual increase to a relatively low maximum growth at 72 h (about 40 % that of Km8 in YPI), indicating glucose repression of inulin utilization as reported in the literature [6, 17]. In YPDI medium, the maximum growth rate of Km8 was about 40 % that of Km7 in YPDI, suggesting this repression affects Km8 more than Km7, although growth decreased for Km7 in YPDI at 72 h compared to Km7 and Km8 in YPI medium. Gao et al. [6] showed that glucose repressed inulinase production at 30 °C, while inulin induced inulinase production. In 1 % inulin medium, no glucose is detected and the maximum growth at 48 h of Km7 was 20 % and of Km8 was 12 % that of these strains in YPI medium. No ethanol was produced by either strain in the 1 % chicory inulin medium. In YPI and YPDI media, Km7 produced more ethanol than Km8, whereas in YPD medium, the production was comparable for the two strains. A comparison of growth, substrate utilization, and ethanol production with *K. marxianus* mutant strains Km7 and Km8 in YPD, YPI, YPDI, and 1 % inulin media to *K. marxianus* strains in the literature is provided in Table 2.

When crude inulin extracted from coffee processing waste was used as substrate, the growth of Km7 cells peaked at about 24 h, whereas the growth of Km8 peaked at about 30 h. In both cases, ethanol production was highest at maximum growth. The amount of inulin present in coffee waste is not well documented and depends on the coffee species, type of processing, and method of extraction. Muthuselvi et al. [26] reported that in spent coffee grounds, a byproduct of the coffee brewing operation that represents 10 % of the total weight of the fresh grain [35], the amount of inulin extracted under normal conditions using hot water at 90 °C for 100 min was 40 µg/mL (0.004 %). In a study using a mixture of coffee pulp and mucilage to simulate coffee waste, the pulp was found to contain 1.3 % fructans and the mucilage was found to contain 3.5 % fructans [12]. The growth and production of ethanol in the crude inulin extract by mutant strains Km7 and Km8 is probably attributable to other molecules in the extract such as proteins and sugars in addition to fructans.

In the zymogram experiments, after staining with PAS, a colorless halo was observed surrounding the region of cell growth of the mutant strains in the inulin-containing gel indicating the absence of inulin, and demonstrating degradation of the inulin in the gel in that area by extracellular inulinase activity of the strains. The reason that Km7 exhibited greater inulinase activity than Km8 may possibly be because microscopic examination showed that the Km7 inoculation resulted in denser colony growth than the Km8 inoculation. The area of cell growth was stained purple because numerous cellular components, such as glycogen, fungal walls, basement membrane, certain epithelial sulfomucins and sialomucins, and neutral mucosubstances, give a positive reaction with PAS. Inulinase activity was quantitated by HPLC measurement of the amount of fructose released by degradation of inulin. Both mutant strains Km7 and Km8 gave a value of 3.7 IU/mL for inulinase activity at 6 h, which is comparable to values reported in the literature [31, 37].

These results suggest that the optimized *K. marxianus* mutant strains NRRL Y-50798 (Km7) and NRRL Y-50799 (Km8) produced by irradiation of *K. marxianus* NRRL Y 1109 also exhibit inulinase activity. Future work will involve an assessment to determine the type of inulinase secreted by each of the strains. These *K. marxianus* mutant strains, especially Km7, have the potential to be used to help remediate inulin-containing fruit and vegetable processing wastes such as those from coffee and tequila production [19, 24]. Inulin and inulin-containing materials also represent renewable and inexpensive and abundant feedstock for bioprocessing to produce valuable fuels and chemicals [3, 18]. The *K. marxianus* mutant strains have potential application in fermentation of inulin-containing plants as well as inulin-containing food and plant waste for an integrated biorefinery platform.

## Electronic supplementary material

Below is the link to the electronic supplementary material.

Fig A1 (Electronic Supplementary Material) Demonstration of inulinase activity in strain Km7 (70 % of full scale); left: inulin-containing gel section with no cell culture added incubated for 5 d at 30 °C and stained with periodic acid solution and Schiff’s reagent (PAS); right: inulin-containing gel section with Km7 cell culture added incubated for 5 d at 30 °C and stained with PAS showing colorless ring around area of cell growth indicating degradation of inulin (PDF 20 kb)
