# Supplementary figures and images for: Growth, ethanol production, and inulinase activity on various inulin substrates by mutant Kluyveromyces marxianus strains NRRL Y-50798 and NRRL Y-50799
Source: J Ind Microbiol Biotechnol. 2016 Apr 29;43:927–39. doi: 10.1007/s10295-016-1771-5 (PMC4902847; doi:10.1007/s10295-016-1771-5)

negative control

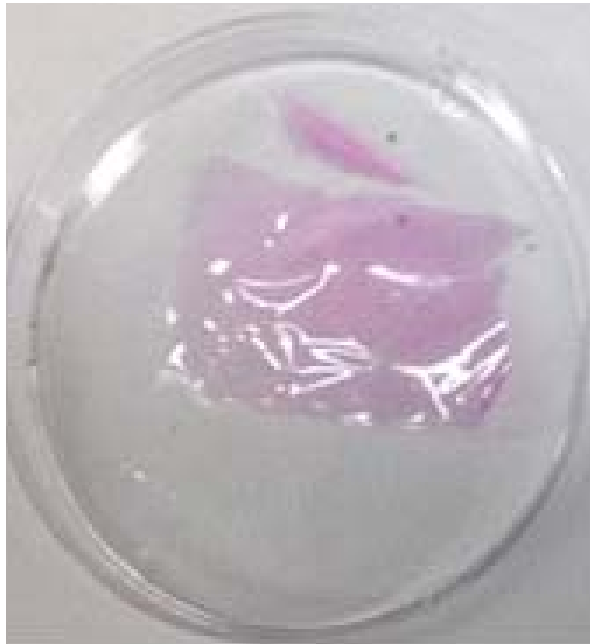

Km7

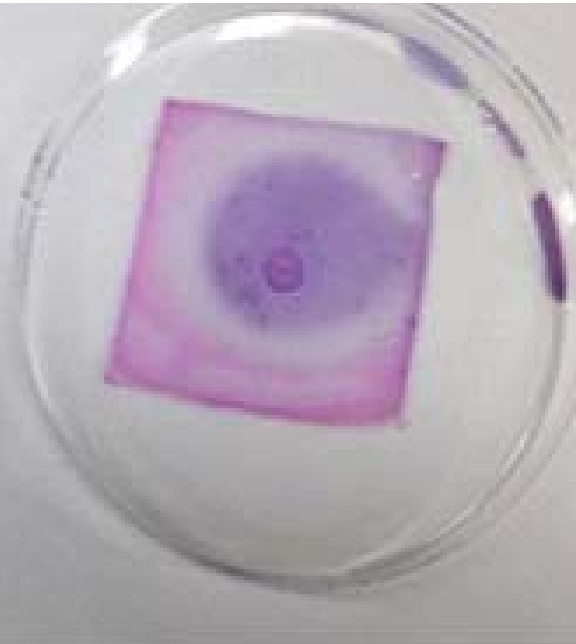

Supplement: Supplementary file 1 — Fig A1 (Electronic Supplementary Material) Demonstration of inulinase activity in strain Km7 (70 % of full scale); left: inulin-containing gel section with no cell culture added incubated for 5 d at 30 °C and stained with periodic acid solution and Schiff’s reagent (PAS); right: inulin-containing gel section with Km7 cell culture added incubated for 5 d at 30 °C and stained with PAS showing colorless ring around area of cell growth indicating degradation of inulin (PDF 20 kb) [file 10295_2016_1771_MOESM1_ESM.pdf]
